# Diagnostic and Management Challenges in Xanthogranulomatous Pyelonephritis: A Case Report

**DOI:** 10.7759/cureus.117382

**Published:** 2026-10-03

**Authors:** Sarah Ahmed, Bushra Aleem

**Affiliations:** 1 Department of Internal Medicine, Whittington Hospital, London, GBR; 2 Department of General Medicine, Whittington Hospital, London, GBR

**Keywords:** bear paw sign, nephrectomy, proteus mirabilis, source control, staghorn calculus, xanthogranulomatous pyelonephritis

## Abstract

Xanthogranulomatous pyelonephritis (XGP) is a rare chronic inflammatory disorder associated with urinary tract obstruction and recurrent infection. Early recognition of XGP is important, as diagnosis is often delayed until significant renal destruction, loss of renal function, and other complications have developed. We report the case of a 36-year-old woman who presented with a three-week history of fatigue, abdominal pain, and markedly raised inflammatory markers. Initial computed tomography (CT) findings were interpreted as pyelonephritis associated with staghorn renal calculi. Following persistent symptoms despite intravenous (IV) antibiotics, multidisciplinary re-review of the imaging identified the characteristic "bear paw" sign of XGP. Ultrasound-guided aspiration isolated *Proteus mirabilis*, allowing targeted antimicrobial therapy. Despite prolonged courses of IV antibiotics as an outpatient, the patient experienced several relapses requiring readmission, particularly following step-down to oral antibiotics. Stone clearance was attempted twice but was unsuccessful due to difficult anatomy, and complications included perinephric abscess formation requiring ultrasound-guided drainage. The choice of definitive management was complicated by preserved renal function in the affected kidney and the patient's preference to avoid nephrectomy. This case highlights the diagnostic challenges of XGP and the need to consider it in patients with persistent or atypical renal infection, particularly with obstructive calculi. Source control is essential for managing infection but may be complicated by challenging anatomy and the implications of nephrectomy.

## Introduction

Xanthogranulomatous pyelonephritis (XGP) is a rare form of chronic pyelonephritis with an estimated incidence of 1.4 cases per 100,000 population annually and occurs more frequently in women [1]. Histologically, it is characterized by replacement of the normal renal parenchyma by lipid-laden macrophages known as xanthoma cells [2]. This destruction of renal tissue commonly leads to loss of renal function, which has often already occurred by the time of diagnosis [3]. Most cases are unilateral, with the disease affecting the entire kidney, although it can also spread to adjacent tissues and the retroperitoneum [4]. Advanced disease may be complicated by abscess or fistula formation. Definitive management is typically total nephrectomy. Partial nephrectomy may be considered in selected patients with focal disease and is performed in approximately 2% of cases. Conservative management has only been described in isolated case reports [5].

## Case presentation

A 36-year-old woman was referred by her general practitioner with a three-week history of fatigue, reduced appetite, and left-sided abdominal pain. Her medical history included an intracranial mesenchymal tumor, which had been treated with retrosigmoid craniotomy and proton beam therapy. Six months prior to this presentation, a surveillance positron emission tomography-computed tomography (CT) had identified left-sided staghorn calculi with associated upper pole calyceal dilatation. As she was asymptomatic from a urinary perspective at the time, no intervention was undertaken.

On admission, she was hemodynamically stable and afebrile. Physical examination was unremarkable except for left upper quadrant and left renal angle tenderness. Laboratory investigations demonstrated a leukocyte count of 14.39 × 10⁹/L (reference range: 3.0-10.0) and C-reactive protein (CRP) of 285 mg/L (0-5). Renal function was normal, with creatinine 50 µmol/L (45-84) and an estimated glomerular filtration rate >90 mL/minute. Urinalysis showed leucocytes and haematuria. CT of the kidneys, ureters, and bladder was initially reported as left-sided pyelonephritis with renal calculi and no evidence of hydronephrosis. Intravenous (IV) ceftriaxone 2 g once daily was commenced, and the patient was referred to the outpatient parenteral antimicrobial therapy (OPAT) service to facilitate early discharge, as she was the primary caregiver for her young daughter. A urology referral was made for stone management.

Despite antibiotic treatment, persistent abdominal pain and raised inflammatory markers, with CRP remaining at 234 mg/L, necessitated continued inpatient management. Repeat multidisciplinary review of the CT identified two upper pole calyceal calculi with associated dilatation, consistent with the characteristic “bear paw” appearance of XGP (Figure 1).

**Figure 1:**
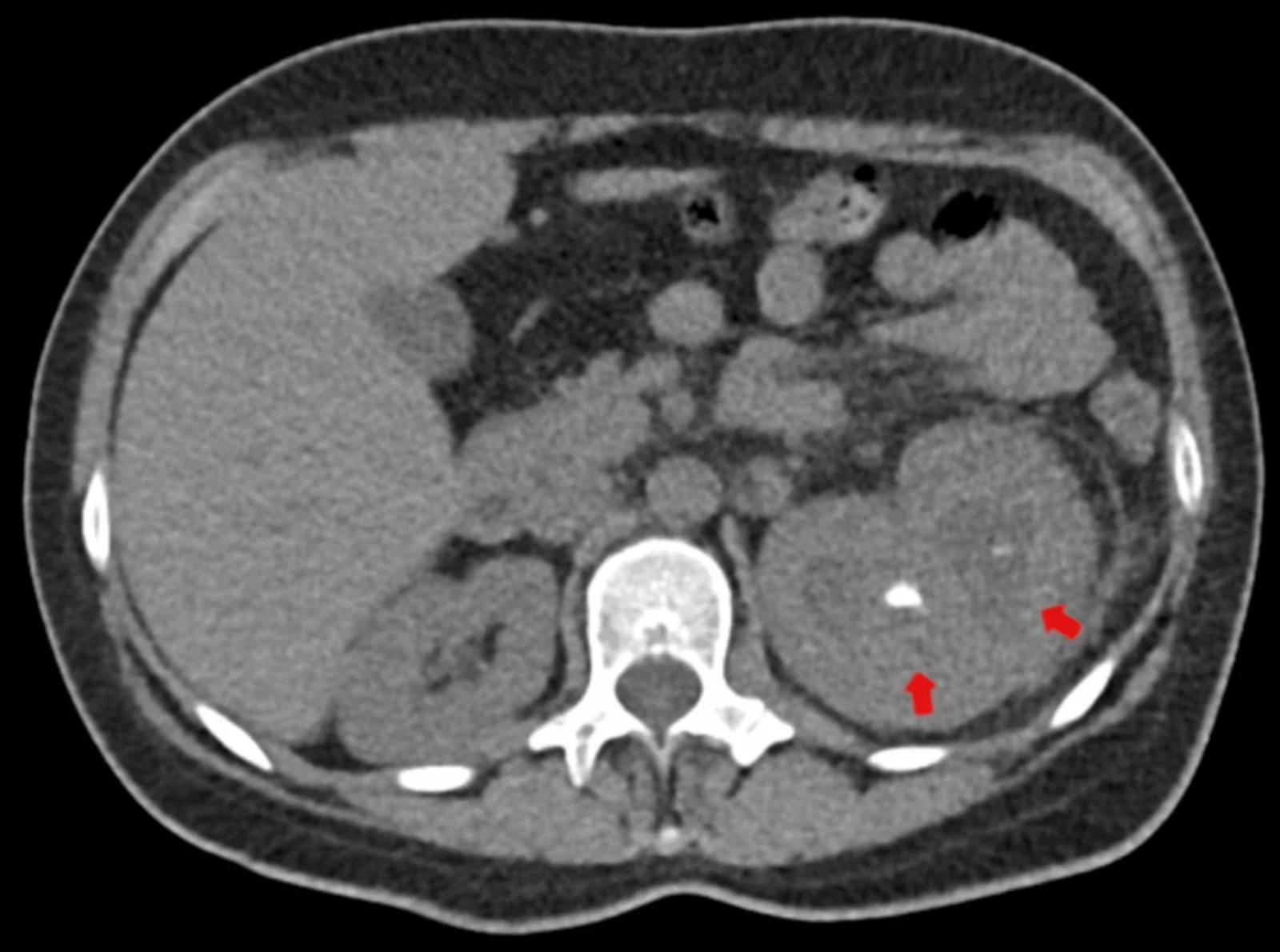
Unenhanced CT demonstrating the bear paw sign Unenhanced CT demonstrating two upper pole calyceal calculi with upper pole calyceal dilatation (red arrows), producing the characteristic "bear paw" appearance of xanthogranulomatous pyelonephritis

Initial blood and midstream urine cultures obtained prior to antibiotic administration showed no bacterial growth. Ultrasound-guided aspiration of the affected calyx was subsequently performed, with four milliliters of green-colored pus aspirated. The sample grew *P. mirabilis*, which was sensitive to ceftriaxone, allowing targeted antimicrobial therapy.

Further imaging supported the diagnosis of XGP. CT urography demonstrated poor drainage of the left upper pole secondary to a 1.4 cm upper pole major calyceal calculus. Technetium-99m (Tc-99m) mercaptoacetyltriglycine renography demonstrated a hypofunctioning left kidney, with essentially absent upper pole function but preserved lower pole function (Figure 2). The patient was discharged under the OPAT service to continue IV antibiotics through a peripherally inserted central catheter.

**Figure 2:**
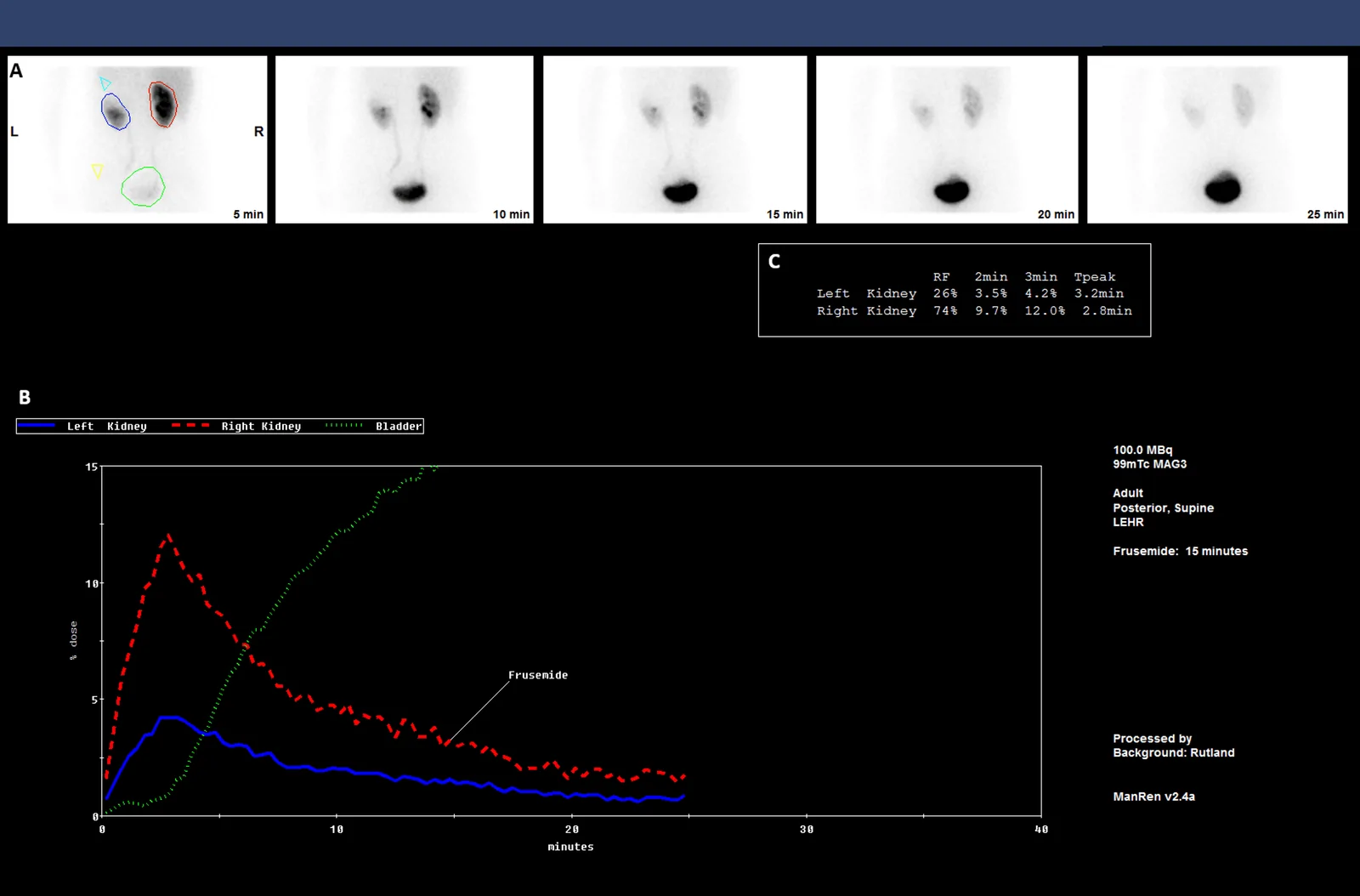
Tc-99m MAG3 renography Tc-99m MAG3 renography. (A) Serial scintigraphic images demonstrating absent upper pole function with preserved lower pole function in the left kidney. (B) Renogram time-activity curves. (C) Differential renal function demonstrating 26% function in the affected left kidney LEHR: low-energy high-resolution; Tc-99m MAG3: technetium-99m mercaptoacetyltriglycine

The patient initially remained on IV ceftriaxone for one month before being stepped down to oral co-amoxiclav 625 mg with additional amoxicillin 500 mg three times daily. Following the transition to oral therapy, she developed recurrent flank pain and rising inflammatory markers, requiring admission and reinitiation of IV ceftriaxone. The subsequent clinical course was characterized by persistent infection, recurrent admissions, and repeated courses of IV antibiotics.

Ureteroscopic stone clearance was recommended following discussion at the urology multidisciplinary team meeting. This was attempted three months after initial presentation but was unsuccessful because the narrow upper pole calyx could not be accessed, and a double-J (JJ) ureteric stent was inserted. The patient reported initial symptomatic relief but, within weeks, experienced a recurrence of infection, with repeat CT urography demonstrating left perinephric and psoas abscesses. The perinephric abscess was successfully drained under ultrasound guidance with good clinical response. A subsequent ureteroscopic attempt at stone clearance again failed. The calculi remained inaccessible within an upper pole calyx containing extensive inflammatory matrix and debris, and the JJ ureteric stent was exchanged. The stent was later removed due to poor tolerance.

The patient remained on prolonged antimicrobial therapy under joint urology and infectious diseases follow-up. Regional multidisciplinary review concluded that definitive management would likely require nephrectomy because adequate source control had not been achieved. However, a Tc-99m dimercaptosuccinic acid single-photon emission computed tomography scan performed eight months after initial diagnosis demonstrated preserved differential renal function of 38% in the affected kidney. Given her young age, improvement in renal function, and preference to avoid nephrectomy, conservative management with suppressive antimicrobial therapy was continued for a further two months. Ultimately, she was counseled that her symptoms were unlikely to resolve without definitive treatment, and she agreed to proceed with nephrectomy.

## Discussion

XGP is a rare, severe form of chronic renal infection that typically results in a nonfunctioning kidney. Its clinical and radiological features can mimic those of most renal inflammatory disorders, including acute pyelonephritis. These nonspecific features, together with its low incidence, can make diagnosis challenging [2].

The precise pathogenesis of XGP remains unclear. However, it is most commonly associated with long-standing urinary tract obstruction and infection. Renal calculi are present in most cases, with staghorn calculi historically reported in 60%-80% of patients [3]. More recent evidence from a systematic review reported staghorn calculi in 48% of cases, with up to 69% demonstrating either renal or ureteric calculi [6]. The most frequently isolated organisms include *P. mirabilis* and *Escherichia coli *[7]. As a urease-producing organism, *P. mirabilis* promotes urinary alkalinization and struvite stone formation [8].

Patients typically present with flank pain that is generally duller than the acute sharp pain of renal colic, as well as fever and weight loss [9]. Imaging plays a central role in diagnosis, assessing disease progression, and identifying associated complications. CT is considered the primary imaging modality for diagnosing XGP [3]. It often demonstrates the characteristic "bear paw" sign [10], which was identified only after multidisciplinary re-review in this case. Histological confirmation, with the pathognomonic finding of lipid-laden foamy macrophages, may be required in diagnostically complex cases [8].

Complications include sepsis, perinephric abscess, psoas abscess, and fistula formation [11]. A nephrocutaneous fistula has also been reported as a rare complication of XGP, illustrating the potential for disease extension beyond the kidney when diagnosis or source control is delayed [12]. Antibiotics are used in all cases but rarely achieve resolution of the chronic infection. In the present case, the patient required prolonged courses of IV antimicrobial therapy. Despite this, she experienced several episodes of clinical deterioration, with recurrent pain and development of perinephric and psoas abscesses.

Source control is essential for definitive management of XGP. Interventional radiological procedures, including percutaneous aspiration and abscess drainage, are increasingly used to manage XGP. Percutaneous aspiration enables direct microbiological sampling of the affected kidney. Cultures obtained from the upper urinary tract have a higher positivity rate than bladder urine cultures and may therefore facilitate targeted antimicrobial therapy [13]. Drainage of abscesses and removal of obstructive calculi are key components in achieving adequate source control [8]. Ultimately, radical nephrectomy with perioperative antibiotic coverage remains the standard of care, particularly when the affected kidney is nonfunctioning [6]. This case illustrates the prolonged and complex clinical course that can accompany conservative management when nephrectomy is deferred.

## Conclusions

XGP should be considered in patients with persistent or atypical pyelonephritis, particularly in the presence of nephrolithiasis and failure to respond to appropriate antimicrobial therapy. This case highlights the challenges of managing XGP when source control is difficult to achieve due to complex anatomy and the chronic inflammatory and fibrotic changes associated with the disease. Although antimicrobial therapy can provide temporary control of infection, definitive resolution typically requires surgical intervention. In patients with preserved renal function, multidisciplinary discussion and shared decision-making regarding nephrectomy are essential to balance infection control, renal preservation, and patient preferences.
